# MicroRNA-27a directly targets *KRAS* to inhibit cell proliferation in esophageal squamous cell carcinoma

**DOI:** 10.3892/ol.2014.2701

**Published:** 2014-11-11

**Authors:** YUZHI JIANG, YUTING DUAN, HAIBIN ZHOU

**Affiliations:** 1 Department of Radiology, Liaocheng People’s Hospital, Liaocheng, Shandong 252000, P.R. China; 2 Department of Thoracic Surgery, Liaocheng People’s Hospital, Liaocheng, Shandong 252000, P.R. China

**Keywords:** microRNA-27a, KRAS gene, esophageal cancer, cell proliferation

## Abstract

MicroRNAs (miRNAs) are a type of small non-coding RNA that negatively regulate gene expression levels by binding to the 3′-untranslated region of specific target mRNAs. To investigate the role of miR-27a in esophageal squamous cell carcinoma (ESCC), TargetScan software was used to predict the target gene of miR-27a. Kirsten rat sarcoma viral oncogene homolog (KRAS), which has been implicated as a regulator of cell proliferation, differentiation and transformation, was identified as a potential target gene of miR-27a and, thus, was the focus of the present study. Luciferase activity in cells transfected with miR-27a mimics was 48% lower when compared with that of the miRNA-negative control. Furthermore, expression levels of the K-ras protein were reduced by ≤50% in cells cotransfected with an expression vector containing miR-27a and miR-27a binding sequences, when compared with the control. The expression level of miR-27a was significantly lower in ESCC cell lines and tissues when compared with healthy esophageal epithelial cells and tissues. However, the expression level of the target gene, KRAS was upregulated and ESCC cell proliferation was significantly inhibited following miR-27a mimic or small interfering K-ras transfection. In conclusion, the present study demonstrated that the expression level of miR-27a was low in ESCC and that miR-27a directly targets the KRAS gene, resulting in inhibited cell proliferation in esophageal cancer.

## Introduction

Esophageal cancer is the sixth most common malignancy worldwide (1,2), responsible for ~482,300 new cases and 406,800 mortalities in 2008 (3–5). The highest incidence rates are in South and East Africa and East Asia, while the lowest rates are in the West and middle of Africa, and Central America (4–6). Esophageal squamous cell carcinoma (ESCC) accounts for >90% of cases of esophageal cancer in the Asia-Pacific region, including China (6–8). ESCC patients are usually diagnosed at a late stage, resulting in a poor prognosis (9–11). Efforts to improve the early detection of ESCC have focused on cytological or endoscopic screening, as well as the application of genetic and epigenetic biomarkers (8–12). Although biomarkers exhibit a high sensitivity, they are unable to conclusively identify which patients are at a low or high risk for disease recurrence. Therefore, there is a requirement for novel prognostic markers and therapeutic targets for ESCC.

MicroRNAs (miRNA) are non-coding RNA molecules (length, 21–25 nt) that inhibit gene expression at the transcriptional and post-transcriptional level by binding to the 3′-untranslated region (3′-UTR) of target mRNAs (12–14). miRNAs bind to partially complementary recognition sequences of mRNA, subsequently causing mRNA degradation or translation inhibition and effectively silencing their target genes (15). Bioinformatic studies indicate that a third of all of the known genes may be regulated by miRNAs. miRNAs have been reported to participate in various important cellular processes, such as apoptosis, cell differentiation and proliferation, tumor suppression, development and metabolism (14,16,17). Recent studies have detected a large number of miRNAs by microarray analysis or other advanced technologies (18–20). Thus, to elucidate the underlying molecular mechanisms associated with ESCC cell proliferation, identification of the regulatory target genes of miRNAs is considered to be critical.

Kirsten rat sarcoma viral oncogene homolog (KRAS), which promotes cell proliferation, was identified as a potential target gene of miR-27a and, thus, was the focus of the present study. The KRAS gene is a member of the mammalian ras gene family and encodes K-ras, a member of the small guanosine triphosphatase superfamily. An activating mutation can be caused by a single amino acid substitution, with the resultant transforming protein identified as an important factor in various malignancies, including lung adenocarcinoma, mucinous adenoma, ductal carcinoma of the pancreas and colorectal carcinoma. The present study examined the correlation between the expression levels of miR-27a and KRAS in ESCC patients and revealed the biological function of miR-27a in ESCC cell lines.

## Materials and methods

### Cell culture

Human ESCC cell lines (TE-1, TE-10, TE-11 and ECA-109) and human esophageal epithelial cells (HEEC), Het-1A, were obtained from the Cell Bank of the China Academy of Sciences (Shanghai, China). All of the human ESCC cell lines were cultured in RPMI-1640 medium (Gibco-BRL, Gaithersburg, MD, USA). HEEC cells were cultured in LHC-9 medium containing 10% fetal bovine serum (HyClone Laboratories, Inc., Logan, UT, USA). All of the media were supplemented with 100 U/ml penicillin and 100 μg/ml streptomycin, and the cells were cultured at 37°C in a 5% CO_2_ atmosphere.

### Construction of recombinant expression vectors

The predicted binding sites on the 3′-UTR of KRAS and 741 bp of the contiguous sequences were cloned into a pGL3 Luciferase^®^ Reporter Vector (Promega Corporation, Madison, WI, USA) and designated as pGL3-Kras-3′-UTR. The mutation plasmid, pGL3-Kras-3′-UTR/mut was also constructed. The coding region sequences of KRAS and binding sequences or site mutation sequences of miR-27a were cloned into the pcDNA3.1(−) plasmid and termed, pcDNA3.1-Kras and pcDNA3.1-Kras/mut, respectively. The primer sequences used in the present study are presented in Table I. To identify miRNAs which are differentially expressed in ESCC and the corresponding adjacent healthy tissues, miRNA Solexa analysis was performed. The expression level of miR-27a was significantly downregulated in ESCC tissues, thus, the target genes of miR-27a were predicted using TargetScan software (http://www.targetscan.org/).

### Dual-Luciferase^®^ Reporter assay

The Dual-Luciferase^®^ Reporter assay system (Promega Corporation) was used to measure the luciferase activity of cells that had been transfected with 400 ng luciferase vector pGL3-Kras-3′-UTR or pGL3-Kras-3′-UTR/mut and either miR-27a mimics or miRNA-negative control (NC). To determine the transfection efficiency, 20 ng pRL-SV-40 (Promega) was cotransfected as the control. Reporter assays were performed at 48 h post-transfection using the Dual-Luciferase^®^ Reporter assay system (Promega Corporation).

### Reverse transcription-quantitative polymerase chain reaction (RT-qPCR)

Total RNA was extracted from the cell cultures using TRIzol reagent (Bio Basic Inc., Toronto, ON, Canada) according to the manufacturer’s instructions. RT was performed using the the PrimeScript^TM^ RT reagent Kit (Takara Biotechnology Co., Ltd., Dalian, China). A cDNA library of miRNAs was synthesized by the QuantiMir™ RT kit (Takara Biotechnology Co., Ltd.). U6 small nuclear RNA and the reference gene, 18S RNA served as the endogenous controls for miRNA and mRNA, respectively. The target genes and controls were treated under the same conditions and analyzed by RT-qPCR using SYBR^®^ Premix Ex Taq™ (Takara Biotechnology Co., Ltd.) according to the manufacturer’s instructions.

### Western blot analysis

Protein for western blot analysis was precipitated according to the standard protocol (15). Equal quantities of protein samples were subjected to SDS-PAGE and transferred to a polyvinylidene fluoride membrane. The membrane was soaked in Tris-buffered saline and Tween-20 (TBST) buffer containing 5% low-fat milk for 60 min with gentle agitation. The membrane was then incubated with monclonal rabbit anti-human c-Kras (1:1,000) and mouse anti-human GAPDH (1:1,000) antibodies (Cell Signaling Technologies, Inc., Danvers, MA, USA) overnight followed by washing with TBST buffer and a further incubation with monoclonal rabbit anti-mouse and mouse anti-rabbit secondary antibodies (1;10,000; Cell Signaling Technologies, Inc.). Finally, an enhanced chemiluminescence reagent kit (Thermo Scientific, Waltham, MA, USA) was used to detect of the protein bands, which were quantified by densitometry (Image Lab™ analysis software; Bio-Rad, Hercules, CA, USA), normalized to GAPDH and expressed as the fold of the control. The primary antibodies used were rabbit anti-c-Kras (1:1,000) and mouse anti-GAPDH (1:1,000). The secondary antibodies were rabbit anti-mouse (1:10,000) and mouse anti-rabbit (1:10,000). All of the antibodies were purchased from Cell Signaling Technology, Inc.

### Cell proliferation

To investigate the effect of miR-27a on cell proliferation, a comparison of the growth rates of ESCC cells transduced with miR-27a or miRNA-NC was performed. Cell growth was determined using a CellTiter 96^®^ Aqueous One Solution Cell Proliferation Assay kit (Promega Corporation). A total of ~5,000 cells were seeded in a 96-well plate 48 h post-transfection and incubated at 37°C for three days. Cell growth was then detected using a 3-(4,5-dimethylthiazol-2-yl) 5-(3-carboxymethoxyphenyl)2-(4-sulfophenyl)-2H-tetrazolium, inner salt (MTS) reduction cell proliferation assay kit every 24 h (0, 24, 48 and 72 h). Absorbance at a wavelength of 490 nm was determined using a microplate reader (OrionL Microplate Luminometer; Titertek-Berthold, Pforzheim, Germany). To investigate whether miR-27a suppresses tumor progression *in vivo*, TE-1 cells were transfected with pEGFP-miR-27a, and G418 was added to the medium and the cells were cultured for one month. The resultant TE-1 cells, which stably expressed miR-27a, were subcutaneously implanted into nude mice to generate tumor xenograft models. Four days after implantation, all of the animals in the control group developed palpable tumors, compared with the mice overexpressing miR-27a, which lacked any detectable tumors.

### Subcutaneous tumor assay

A total of five six-week-old BALB/c-A nude mice were purchased from the animal center of the Cancer Institute of the Chinese Academy of Medical Science (Beijing, China). All experimental procedures were conducted according to the regulations, and the internal biosafety and bioethics guidelines of Liaocheng Hospital (Liaocheng, China). The TE-1 subcutaneous model was established as previously described (22). TE-1 cells stably expressing miR-27a were injected into the mice. Treatment was conducted at four-day intervals until completion of the experiment. The tumor volume was measured with a caliper every four days and the following formula was used: volume (mm^3^) = (length × width^2^)/2. At the end of a 24-day observation period, the mice were sacrificed, and the tumor tissues were collected for formalin fixation and preparation of paraffin-embedded sections for immunohistochemical analysis.

### Statistical analysis

Results are expressed as the group means ± standard error of the mean and were analyzed using GraphPad Prism 5 software (GraphPad Software, Inc., La Jolla, CA, USA) using unpaired t-tests for two-group comparisons and one-way analysis of variance for three or more group comparisons. P<0.05 was considered to indicate a statistically significant difference.

## Results

### miR-27a directly targets the KRAS gene by interaction with the 3′-UTR

**Figure 1:**
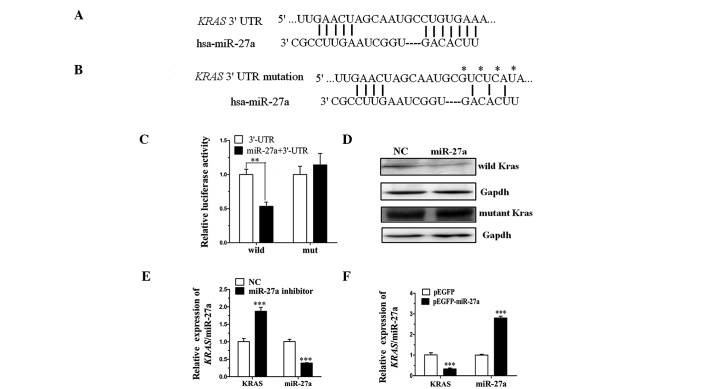
miR-27a directly targets the KRAS gene by interacting with its 3′-UTR. (A) miR-27a binding sequence in the 3′-UTR of KRAS; and (B) a mutation of the KRAS 3′-UTR generated in the site complementary to the seed region of miR-27a. ^*^Indicates the mutant nucleotide. (C) Relative luciferase activity (normalized control group activity) of HEK-293T cells tranfected with pGL3-Kras-3′-UTR or pGL3-Kras-3′-UTR/mut, and miRNA-NC or miR-27a mimics. (D) Western blot analysis indicating the expression of K-ras wild-type and mutant K-ras protein following miR-27a mimic or miRNA-NC transfection. GAPDH served as an internal control. (E and F) Relative expression of miR-27a and KRAS was detected by reverse transcription-quantitative polymerase chain reaction following miR-27a inhibitor or pEGFP-miR-27a transfection in TE-10 or TE-1 cells, respectively. ^*^P<0.05 vs. NC transfected group; ^**^P<0.01; ^***^P<0.001. U6 small nuclear RNA and 18S RNA served as the internal controls in A and C, and B and D, respectively. Each assay was performed in triplicate. miRNA, microRNA; KRAS, Kirsten rat sarcoma viral oncogene homolog; UTR, untranslated region; NC, negative control.

TargetScan (http://www.targetscan.org) and PicTar (http://pictar.mdc-berlin.de) are types of software broadly used online to predict miRNA targets. The present study utilized TargetScan and PicTar to predict the target miRNA of KRAS (Fig. 1A and B) and demonstrated that miR-27a targets the 3′-UTR of KRAS. To clarify this, pGL3-Kras-3′-UTR containing the miR-27a binding sequences and pGL3-Kras-3′-UTR/mut were constructed. Analysis of luciferase activity demonstrated that the activity of miR-27a mimics that were cotransfected with pGL3-Kras-3′-UTR was significantly more inhibited when compared with the miRNA-NC (P<0.01). However, the activity of miR-27a mimics that were cotransfected with pGL3-Kras-3′-UTR/mut exhibited no significant difference when compared with the miRNA-NC (Fig. 1C). Thus, the luciferase activity assay indicated that the mutated 3′-UTR affected the binding of miR-27a.

Furthermore, to investigate whether miR-27a affects KRAS expression at the transcriptional and translation levels, two types of expression plasmid were constructed. The expression plasmids, pcDNA3.1-Kras and pcDNA3.1-Kras/mut contain the coding regions and 3′-UTR sequence of KRAS, however, the pcDNA3.1-Kras/mut contains the mutated miR-27a binding sequences. Western blot analysis demonstrated that the expression level of miR-27a cotransfected with pcDNA3.1-Kras was markedly lower when compared with the miRNA-NC. However, no significant difference was identified between miR-27a mimics and miRNA-NC cotransfected with pcDNA3.1-Kras/mut (Fig. 1D). Finally, the endogenous KRAS was detected by RT-qPCR following transfection of the miR-27a inhibitor into TE-10 cells or pEGFP-miR-27a into TE-1 cells. The expression levels of miR-27a and KRAS demonstrated negative correlation (Fig. 1E and F). These data indicated that miR-27a directly targets KRAS in ESCC by binding to the 3′-UTR of the KRAS gene.

### Expression level of miR-27a and KRAS in ESCC cell lines and patient tissue samples

**Figure 2:**
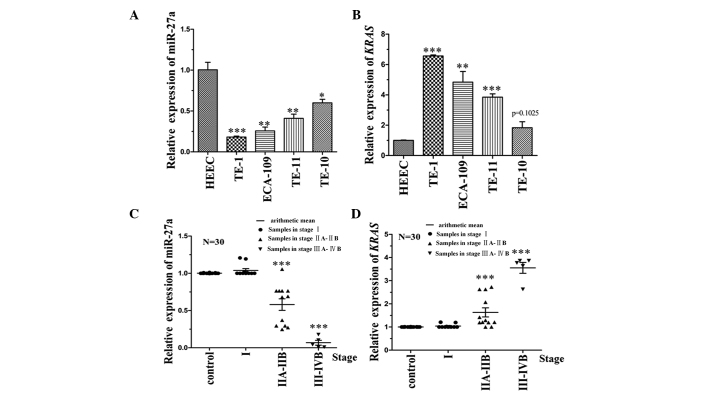
Expression levels of (A) miR-27a and (B) KRAS in various ESCC cell lines. The expression level of each gene is the fold-change relative to the expression level of HEEC (the control). Expression levels of (C) miR-27a and (D) KRAS in 30 lung cancer patient tissues. U6 small nuclear and 18S RNA served as the internal controls in A and C, and B and D, respectively. Data was obtained by reverse transcription-quantitative polymerase chain reaction and each assay was performed in triplicate. ^*^P<0.05; ^**^P<0.01; ^***^P<0.001. miR, microRNA; KRAS, Kirsten rat sarcoma viral oncogene homolog; ESCC, esophagus squamous cell carcinoma; HEEC, human epithelial esophageal cells.

To identify the correlation between miR-27a and KRAS expression levels in ESCC cells, RT-qPCR analysis was performed on four different ESCC cell lines (TE-10, TE-11, TE-1 and Eca-109) and one healthy HEEC line (Het-1A), which served as the control. The data demonstrated that the expression level of miR-27a was negatively correlated with KRAS (Fig. 2A and B). The expression level of miR-27a and its target, KRAS, were also detected in 30 patient tissue samples. The clinicopathological characteristics of the 30 patients are indicated in Table II. The expression level of KRAS in stage III–IVB tumor samples (23) was significantly higher than that in stage I tissue samples (P<0.001). The corresponding target gene (miR-27a) was negatively correlated with the KRAS expression level (Fig. 2C and D). Thus, the results of the present study indicated that miR-27a affects KRAS expression levels.

### miR-27a inhibits cell proliferation by reducing the expression level of KRAS in ESCC

**Figure 3:**
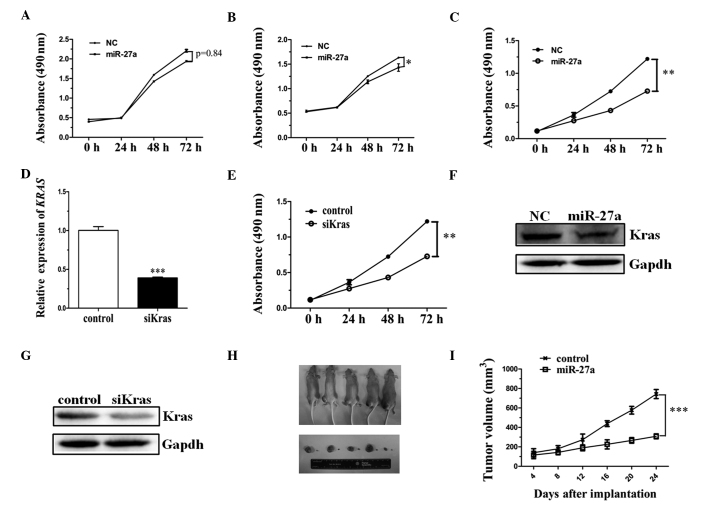
miR-27a inhibits cell proliferation by reducing the expression of KRAS in ESCC cell lines and animal models. (A) TE-11, (B) ECA-109 and (C) TE-1 cells were transfected with miR-27a or miRNA-NC. Following reseeding, cell numbers were measured using an MTS assay kit at 0, 24, 48 and 72 h. (D) KRAS expression was detected by reverse transcription-quantitative polymerase chain reaction following siK-ras transfection into the TE-1 cell line or siNC as a control. (E) TE-1 cells were transfected with siK-ras or siNC, using the same treatment as the miR-27a mimic transfection. (F) Western blots demonstrating the expression of K-ras following miR-27a mimic or or miRNA-NC transfection. (G) Western blots demonstrating the expression of K-ras following siK-ras or siNC transfection. GAPDH served as the internal control. (H) Nude mice were photographed 24 days after injection with TE-1 cells transfected with pEGFP-miR-27 or pEGFP as a control. (I) Tumor size was measured every four days and tumor growth curves were generated. Each assay was performed in triplicate.^*^P<0.05, ^**^P<0.01; ^***^P<0.001. miRNA, microRNA; KRAS, Kirsten rat sarcoma viral oncogene homolog; ESCC, esophagus squamous cell carcinoma; NC, negative control; siK-ras, small interfering K-ras; siNC, siK-ras negative control.

To investigate whether miR-27a functions as a tumor suppressor, an MTS assay was performed to detect cell viability. ECA-109, TE-11 and TE-1 cell lines transfected with miR-27a were observed to grow at a reduced rate when compared with those cells transfected with mRNA-NC (Fig. 3A–C). Thus, the results of the present study indicated ectopic miR-27a expression may inhibit the proliferation of ESCC cell lines.

To identify whether the downregulation of KRAS alone inhibits the proliferation of the TE-1 cell line, a small interfering (si)K-ras expression vector was constructed. Results from the present study indicated that the level of KRAS expression was significantly reduced when compared with the control (Fig. 3D). MTS was subsequently performed to detect the cell viability following siK-ras expression vector transfection. The results were consistent with cell viability following miR-27a mimic transfection, when compared with the control (Fig. 3E). Furthermore, the expression level of the endogenous K-ras protein was detected by western blot analysis, indicating that the expression level of KRAS was obviously reduced in miR-27a-transfected and siK-ras vector-transfected ESCC cells (Fig. 3F and G). Thus, miR-27a promotes cell proliferation by reducing the expression of KRAS in ESCC cell lines.

Upon termination of the experiment, the tumor volume demonstrated that the tumor growth rate was substantially lower in mice implanted with the cells overexpressing miR-27a compared with the control mice (Fig. 3H). On the 24th day after implantation, the mean tumor volume of the miR-27a overexpression group (270 mm^3^) was significantly smaller than that of the control group (710 mm^3^) (Fig. 3I; P<0.001).

## Discussion

miRNAs are key in the regulation of cell proliferation, apoptosis and other important cellular processes. The role of miRNA in each specific cell line is dependent on the specific target gene of the miRNA (15,24–26). Thus, a single miRNA may exhibit an opposite role in a different cell line. Therefore, identifying the target gene of miRNA is considered to be critical. miR-27a was identified to be downregulated in acute leukemia cell lines and primary samples when compared with hematopoietic stem-progenitor cells (HSPCs), which indicates that miR-27a may exert a tumor suppressor-like action in acute leukemia, possibly via the regulation of apoptosis (27–30). However, miR-27a expression was upregulated during C2C12 myoblast proliferation, indicating that miR-27a may promote myoblast proliferation by targeting myostatin (31–36). The present study investigated the variation in miRNA expression levels in ESCC. The expression of miR-27a was significantly downregulated when compared with a healthy animal model and human esophageal tissues, which indicated that miR-27a may function as a tumor suppressor in ESCC. Furthermore, an MTS assay identified the role of miR-27a in ESCC.

miR-27a may target a number of genes in ESCC. TargetScan software was used to predict the target gene of miR-27a. The KRAS gene was selected as the potential target for further investigation. Previously, KRAS has been identified as a possible target for cancer therapeutics, due to its activation driving a number of traits associated with tumor cells, in particular cell growth and proliferation. KRAS is a target of miR-27a in tumors, particularly in ESCC, and downregulation of the KRAS oncogene may provide a novel treatment strategy for cancer patients by attenuating tumor growth. The Dual-Luciferase^®^ Reporter assay indicated that the target gene (KRAS) may be directly targeted by miR-27a and western blot analysis consistently indicated that the endogenous K-ras protein is inhibited by miR-27a. Furthermore, the proliferation of the TE-1 cell line was significantly inhibited upon siK-ras and miR-27a transfection.

In conclusion, the present study identified that miR-27a functions as a tumor suppressor in ESCC, by direct targeting of the KRAS gene.

## Figures and Tables

**Table I:** Primer sequences for the construction of the recombinant expression vectors used in the present study.

Plasmid	Primer sequence	Restriction enzyme
pGL3-Kras-3′-UTR	Forward: GAGCAAAGATGGTAAAAAGA	*Xba*I
	Reverse: TAAATATAGCCCCAAAATGG	*Eco*RV
pGL3-Kras-3′-UTR/mut	Forward: AACTAGCAATGCGTCTCATAAAGAAACTGAATACCTAAGATTTCTGTC	
	Reverse: GACAGAAATCTTAGGTATTCAGTTTCTTTATGAGACGCATTGCTAGTT	
pcDNA3.1-Kras	Forward: ATGACTGAATATAAACTTGTGGTAG	*Xho*I
	Reverse: ACTAGATAAAACACAGAATAGGGAT	*Eco*RV
pcDNA3.1-Kras/mut	Forward: AACTAGCAATGCGTCTCATAAAGAAACTGAATACCTAAGATTTCTGTC	
	Reverse: GACAGAAATCTTAGGTATTCAGTTTCTTTATGAGACGCATTGCTAGTT	
pEGFP-miR-27a	Forward: AAGTTGCTGTAGCCTCCTTGTCC	*Xba*I
	Reverse: CCCACTCACCCACCTATCTATGC	EcoRI

UTR, untranslated region.

**Table II:** Data of the esophageal cancer patients.

				TNM staging		
				
No.	Gender	Age, years	Comprehensive stage	Tumor	Lymph node	Metastasis
1	M	56	IA	T1B	N0	M0
2	M	56	IA	T1B	N0	M0
3	M	55	IB	T2B	N0	M0
4	F	48	IIA	T1B	N1	M0
5	M	86	IA	T1B	N0	M0
6	F	57	IA	T1B	N0	M0
7	M	62	IIA	T1B	N1	M0
8	F	54	IIA	T1B	N1	M0
9	F	59	IIIA	T2A	N2	M0
10	F	63	IIB	T2A	N1	M0
11	M	72	IB	T2B	N0	M0
12	M	61	IIA	T1B	N1	M0
13	M	78	IIB	T2A	N1	M0
14	M	66	IIA	T2A	N1	M0
15	F	45	IB	T2A	N0	M0
16	F	63	IIA	T1B	N1	M0
17	F	52	IIIA	T3	N2	M0
18	F	54	IB	T2B	N0	M0
19	M	58	IIIA	T3	N2	M0
20	F	64	IB	T2A	N0	M0
21	F	67	IIA	T2A	N1	M0
22	M	52	IIA	T2A	N1	M0
23	M	80	IB	T2	N0	M0
24	M	56	IIIB	T4	N2	M1
25	F	63	IIA	T2A	N1	M0
26	F	54	IIA	T2A	N1	M0
27	M	71	IIA	T2A	N1	M0
28	M	72	IV	T1	N0	M1
29	F	68	IA	T1	N0	M0
30	M	76	IIA	T2	N1	M0

TNM staging according to the National Comprehensive Cancer Network guidelines (21). TNM, tumor node metastasis; M, male; F, female.
